# Dysphagia and Body Composition in Cornelia de Lange Syndrome

**DOI:** 10.3390/biomedicines12112551

**Published:** 2024-11-08

**Authors:** Aleksandra Mędza, Aleksandra Cieszko, Małgorzata Gliwa, Michał Brzeziński, Jolanta Wierzba, Agnieszka Szlagatys-Sidorkiewicz, Katarzyna Sznurkowska

**Affiliations:** 1 Department of Pediatrics, Pediatric Gastroenterology, Allergology and Nutrition, Copernicus Hospital, Nowe Ogrody 1-6, 80-803 Gdansk, Poland; zdobylak.a@gmail.com (A.C.); mggliwa@gmail.com (M.G.); 2 Department of Pediatrics, Pediatric Gastroenterology, Allergology and Nutrition, Medical University of Gdansk, Nowe Ogrody 1-6, 80-803 Gdansk, Poland; brzezinski@gumed.edu.pl (M.B.); agnieszka.szlagatys-sidorkiewicz@gumed.edu.pl (A.S.-S.); katarzyna.sznurkowska@gumed.edu.pl (K.S.); 3 Department of Internal and Pediatric Nursing, Medical University of Gdansk, 80-210 Gdansk, Poland; jolanta.wierzba@gumed.edu.pl

**Keywords:** dysphagia, bioelectric impedance, CdLS, body composition, phase angle, PEDI-EAT-10

## Abstract

Background/Objectives: Limited research had investigated nutritional status in patients with Cornelia de Lange Syndrome (CdLS) (OMIM 122470, 300590, 300882, 610759, 620568 and 614701). Body composition assessed via bioelectric impedance (BIA) is a particularly under-explored issue. In this cross-sectional study, we hypothesize that body composition imbalance is frequent in CdLS and may be associated with dysphagia. We aimed to determine dysphagia prevalence in CdLS. Dysphagia may be a sign or a complication of GERD (gastroesophageal reflux disease), which is the most frequent gastroenterological disorder in CdLS patients; Methods: Fourteen Polish patients with a clinical or genetic diagnosis of CdLS were included in the study. We performed body composition analysis via bioelectric impedance taking into account the phase angle (PhA) and Body Cell Mass (BCM) in patients who cooperated and were able to sit still. The patients’ caregivers completed the pediatric version of the Eating Assessment Tool (PEDI-EAT-10). Based on the questionnaire scoring, we divided the patients into dysphagic and non-dysphagic groups. Body compartments of those two groups were compared. Statistical correlations between PhA and the PEDI-EAT-10 score were calculated; Results: Eleven of the fourteen CdLS patients had abnormalities in the BIA analysis in terms of fat mass (FM), fat free mass (FFM) and skeletal muscle mass (SMM). Six patients had excessive FM and four patients were deficient in FM. Two had deficiency in FFM and two had excessive FFM. We noted prevalence of dysphagia at 28.57%, with four patients having an PEDI-EAT-10 score higher or equal to 3, categorized as dysphagic. The dysphagic and non-dysphagic groups were not significantly different in terms of the proportion of patients with FM, FFM, SMM and BCM in the small cohort presented here. A statistically significant inverse correlation was found between the PhA and PEDI-EAT-10 score (rho = −0.72; *p* = 0.003); Conclusions: CdLS patients require investigation for dysphagia and nutritional status imbalance, as they are both frequent in this syndrome. The most prevalent are abnormalities in FM, both excess and deficit. PhA deviations observed in the bioimpedance study deepen with the severity of dysphagia. These findings require further investigation in a larger cohort.

## 1. Introduction

Cornelia de Lange Syndrome (OMIM 122470, 300590, 300882, 610759, 620568 and 614701) is a multisystem genetic disorder, caused by variants in any genes of the cohesion protein complex. The cardinal features, considered to be the most characteristic for CdLS, include pre- and postnatal microsomia and microcephaly, dysmorphic face features with synophrys, thick eyebrows, short nose, concave nasal ridge, upturned nasal tip, long or smooth philtrum, thin upper lip or downturned corners of the mouth, and limb malformations. Others, such as global developmental delays or intellectual disability and autistic-like behaviors, small hands or feet, short fifth finger, and hirsutism are suggestive features, which contribute to the diagnosis, but are less specific [1].

The first international consensus on the diagnosis and management of CdLS describes the classic and non-classic CdLS phenotypes using a scoring system consisting of a combination of the signs and symptoms listed above [1]. The presentation of the disorder is variable, and the phenotypes attributed to Cornelia de Lange Syndrome can be characterized as located on a spectrum. Independently of the disease phenotype, about 65–75% of CdLS patients are diagnosed with gastroesophageal reflux disease (GERD), which is a frequent and severe gastroenterological problem in CdLS [2,3]. A typical symptom of GERD is dysphagia [4]. According to Kline et al., 36% of adult CdLS patients reported dysphagia symptoms [3], but the reasons for dysphagia were not provided. Dysphagia is characterized by difficulty in swallowing, which may occur in the oral, pharyngeal, or esophageal phase and it may affect the swallowing of liquid and solid foods. Beyond prolonged mealtimes and the discomfort experienced by the patients, dysphagia is associated with serious complications which include malnutrition and impaired development, choking, and recurrent pneumonia. In CdLS patients, respiratory problems including aspiration/reflux and pneumonias are the most common primary reason of premature death, accounting for 31% of the causes of death [5]. Other causes of early death in that syndrome include gastrointestinal diseases, predominantly ileus, congenital anomalies, neurological causes and sepsis.

Currently there are no scientific studies reporting body composition measured via bioelectrical impedance analysis in individuals with CdLS. Estimating body compartments involved in the regulation of the whole body energy and metabolism provides us with a more sophisticated view on nutritional status and diseases [6]. This includes patients with normal weight or BMI (Body Mass Index), for whom excess fat mass might pose a cardiometabolic disease risk or having a lean mass deficit might lead to impaired body function [7]. Body composition assessed using BIA may be described using the two-compartment model, which estimates the fat mass (FM) and fat free mass (FFM). While it is possible to calculate the percentage of each compartment, it is better to use tissue mass, because a low FM% may reflect either a high FFM% or a low FM%. More accurate data can be obtained from the fat mass index (FMI) or fat free mass index (FFMI), where tissue mass is adjusted for height, by dividing FFM or FM by height squared. Bioelectric impedance analysis estimates FFM and FM by measuring the impedance or resistance to electrical current as it flows through the body’s water pool with dissolved electrolytes—the major conductors of electrical current. Thus, BIA starts with measuring the total body water. Further, analyzers use total body water to predict FFM with an assumption that 73% of the body’s FFM is water in healthy individuals. This is one of the limitations of this method and newer analyzers try, in various ways, to reduce the risk of error associated with this assumption [8]. Finally, FM is calculated from the assumption that total body mass is FM and FFM [9]. This method also makes it possible to measure the bioelectrical phase angle (PhA), which describes nutritional status in children [10]. It assesses the relationship between resistance (Rz) and reactance (Xc) and characterizes body cell condition [11]. Phase angle is an indicator of the integrity of cell membranes, reflecting the efficiency of energy processes and proteolysis. PhA serves as a prognostic or risk factor [12,13,14] for many different medical conditions. Phase angle values decrease in conditions with inflammation, malnutrition and prolonged physical inactivity [15]. For example, low PhA values have been reported in sarcopenia, reflecting poor muscle status [16,17,18].

Phase angle has been shown to correlate with body weight and arm circumference. Since arm muscle circumference serves as an indicator of skeletal muscle mass, it can be inferred that the phase angle also reflects body cell mass (BCM) in children and adults. BCM and PhA are co-dependent and indicate patients’ health condition [19,20]. Body cell mass (BCM) reflects the functional mass of the body, with metabolically active components. To obtain BCM, one must consider the FFM without extracellular water and bone mineral mass.

Our goal was to assess the prevalence of dysphagia and to describe body composition in CdLS patients. This study hypothesized that body composition imbalance is prevalent in CdLS patients and may be associated with dysphagia.

## 2. Materials and Methods

Initially, our study covered 24 patients with a genetic or clinical diagnosis of Cornelia de Lange Syndrome who participated in the annual meeting of Polish CdLS families in 2023 or came to the Department of Pediatrics, Pediatric Gastroenterology, Allergology and Nutrition, Medical University of Gdansk, to participate in the study. All patients were diagnosed using a clinical evaluation of phenotypic features according to the international consensus statement [1], and 13 were confirmed to have genetic mutations typical for CdLS: NIPBL-10, SMC1A-1, SMC3-1 and DDX23-1. Although DDX23 has not been listed in the diagnosis consensus [1], this individual typical fenotype for CDLS has been associated with DDX23, which has been described and published [21]. The parents of one child did not consent to the genetic test.

The 10 patients of the initial 24 excluded from the study or subsequent analysis included 4 patients who did not cooperate, could not stay still and did not tolerate the presence of the analyzer electrodes on their body, so it was not possible to measure bioimpedance. However, we allowed the results from the patients who moved once, not very vigorously, during the analysis. Three patients were excluded due to analyzer limitations (under 3 years old, weighed under 10 kg or/and were under 95 cm tall); one patient did not complete the questionnaire, one was fed via a PEG tube and had undergone antireflux surgery; and one patient had an incorrect height measurement entered in the BIA analysis (patient’s code—D2).

Finally, the study group included 14 participants, 6 males and 8 females. The median age of the patients was 12.5 years, mean 17.53, and a range of 5–42 years old. Nine patients had been diagnosed with GERD in the past, but only five were undergoing treatment with Proton Pump Inhibitors (PPIs) at the time of the study.

The assessed patients were divided into two groups: with and without dysphagia symptoms. This was accomplished using the pediatric version of the Eating Assessment Tool EAT-10 through the caregivers [22]. This is considered to be a quick, easy-to-complete, non-invasive, inexpensive screening tool to preliminarily assess dysphagia severity before performing a more specific examination. The questionnaire contains 10 statements:

My child does not gain weight due to his/her swallowing problem.
The swallowing problem of my child interferes with our ability to go out for meals.
Swallowing liquids takes extra effort for my child.
Swallowing solids takes extra effort for my child.
My child gags during swallowing.
My child acts like he/she is in pain while swallowing.
My child does not want to eat.
Food sticks in my child’s throat and my child chokes while eating.
My child coughs while eating.
Swallowing is stressful for my child.

Answers are based on the caregivers’ perception and are rated from 0 to 4 (0: no symptom; 4: severe symptom), with a possible total score ranging from 0 to 40.

Firstly, we measured each patient’s weight and height using a Seca electric scale with a stadiometer. With individuals who were not able to stand up, we used a measuring mat.

Secondly, bioimpedance measurements were performed on patients who cooperated and were able to remain still, mostly in a sitting position, with one patient laying down. The BIA measurements were taken in fasting patients and after urination. The analysis lasted 1 min and was performed using the Inbody S10 8-point tetrapolar touch electrode system (2 left foot electrodes, 2 right foot electrodes, 2 left hand electrodes, 2 right hand electrodes). With patients with anatomical defects affecting their fingers, self-adhesive electrodes were used instead of touch electrodes. The InBody S10 analyzers are distinguished from other devices by the fact that the size of the hand has no effect on the test result, which is important in children with hand anomalies. According to the manufacturer’s instructions, the InBody S10 does not use empirical equations that incorporate age and sex to determine body composition; these data are only used to provide normal ranges for each test subject. The InBody S10 uses multi-frequency current (5, 50, 250, 500 and 1000 hHz), which enables accurate measurement of intra- and extracellular water.

We determined parameters such as fat mass (FM), fat free mass (FFM), skeletal muscle mass (SMM) and body cell mass (BCM), analyzing the numbers of patients in whom they occurred below, above or within the range of the individual norms calculated by the InBody S10 analyzer in kilograms. Phase angle (PhA), expressed in degrees, was also determined.

The body composition parameters were compared between patients who were dysphagic and without dysphagia using the chi-squared test with Fisher’s exact test. Finally, we calculated Spearman’s correlation coefficient between PhA and the PEDI-EAT-10 scores. Statistical analyses were performed using IBM SPSS Statistics 29. A threshold of α = 0.05 was used as the level of significance.

## 3. Results

The positive answers to the statements in the PEDI-EAT-10 questionnaire rate the severity of the problems from 0 to 4, with 0 points indicating no problem and 4 points indicating a severe problem.

Out of the 14 patients included in the study, 4 (28.57%) obtained a PEDI-EAT-10 score equal or greater than 3 and they are later described as the “dysphagia group”. Their scores were 5, 8, 10 and 20 points. The remaining 10 were classified as non-dysphagic, with 6 obtaining a 0-point score on the PEDI-EAT-10.

For patients under 18 years old, we used centile grids specific for CdLS [23] to assess height and weight, and we present these data in Table 1.

Of all CdLS patients included in the study, 78.57% (11 of 14) had abnormal BIA results, with the most common being excessive fat mass in 6 out of the 14 (42.86%) patients.

**Figure 1:**
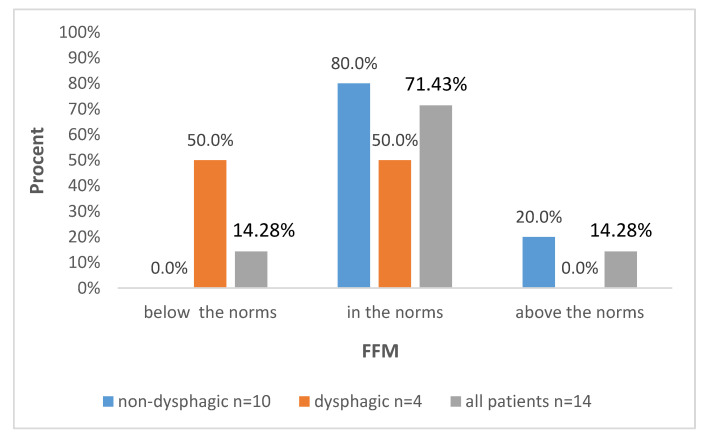
Distribution of FMM levels according to the presence of dysphagia and in the whole studied group.

**Figure 2:**
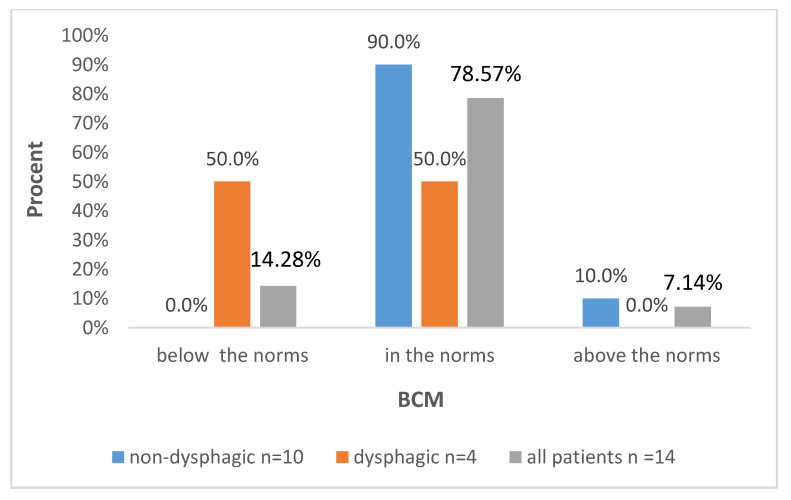
Distribution of BCM levels according to the presence of dysphagia and in the whole studied group.

**Figure 3:**
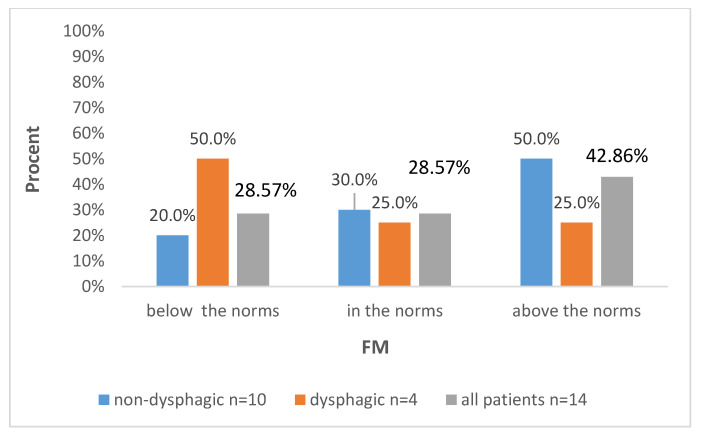
Distribution of FM levels according to the presence of dysphagia and in the whole studied group.

**Figure 4:**
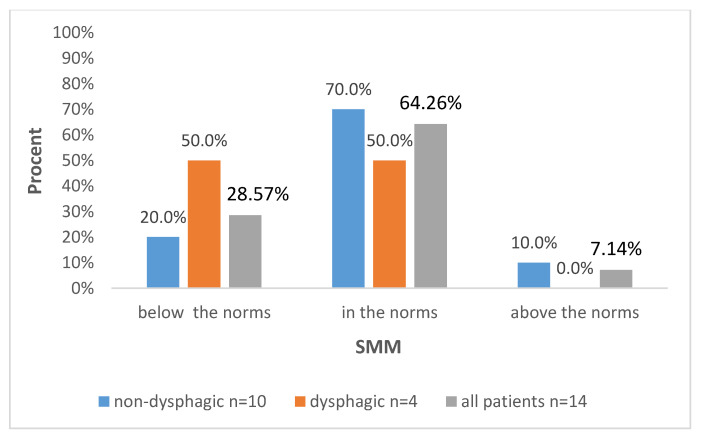
Distribution of SMM levels according to the presence of dysphagia and in the whole studied group.

Further data are presented in Figure 1, Figure 2, Figure 3 and Figure 4, with the percentages of patients being below, above or in the normal range regarding the analyzed parameters with regard to all included patients and also divided into subgroups: dysphagic and non-dysphagic.

We compared our two groups, dysphagic and non-dysphagic, in terms of the number of patients being under, over or in the normal range for FM, FFM, SMM, and BMC. Statistical analysis showed that the dysphagic and non-dysphagic groups were not different in terms of the number of patients within the normal range for fat free mass (*p* = 0.131), FM (*p* = 0.76), SMM (0.664) or BMC (*p* = 0.066). The value of Cramér’s V indicated a moderately strong relationship between the variables for FM (Vc = 0.31) and SMM (Vc = 0.32) and a strong relationship for FFM (Vc = 0.66) and BCM (Vc = 0.65).

Table 2 and Table 3 contain data for all patients divided into two groups, dysphagic and non-dysphagic.

We have found a statistically significant inverse correlation between the phase angle (PhA) and PEDI-EAT-10 score with Spearman’s r of −0.72; *p* = 0.003.

## 4. Discussion

To our knowledge, this is the first study to investigate dysphagia and body composition in Cornelia de Lange Syndrome patients. Since this syndrome was first described in 1916, there has been little research relating to nutritional status. Most gastroenterology studies refer to dysphagia in gastroesophageal reflux disease but do not distinguish it as a separate problem caused by other, non-reflux, conditions.

Our results showed that the dysphagic and non-dysphagic groups were not significantly different in terms of the proportion of patients with FM, FFM, SMM and BCM. However, it should be underlined that results were obtained from statistical analysis on a very small study group. The contingency table of FFM in the dysphagia and non-dysphagia groups shows that among children with dysphagia, 50.0% each had normal and below-normal scores on the FFM parameter, while in the other group, 80.0% of the subjects had normal and 20.0% had above-normal scores. Similarly, the cross-tabulation analysis of BMC shows that among those with dysphagia, 50.0% scored below-normal and normal in terms of BMC, while in the group without dysphagia, 90.0% had BMC levels within normal and 10.0% above normal ranges. A more in-depth statistical analysis, taking into account the index of effect size (Vc = 0.66 for FFM and Vc = 0.65 for BCM), indicates possible differences between the compared groups that could be demonstrated in the study on a larger group. Thus, it can be suspected that patients with dysphagia obtain lower values of the FFM and BCM parameter compared to children without dysphagia. These results need to be verified on larger study cohort.

Nutritional status in CdLS is an underexplored issue, yet it is of potentially significant clinical importance. For example, an Italian study of CdLS patients showed that qualitative and quantitative abnormalities were present more frequently than expected [24]. In that study group, only 15/62 (24%) patients had an optimum caloric intake, whereas the intake was low in 27/62 (43%) subjects. In the same study, blood tests revealed a low iron level in 22/73 (30%) patients and 25(OH)D deficiency in 49/73 (67%).

Matute Llorente et al. investigated body compartments in CdLS [25], describing nine patients whose body composition was evaluated using dual-energy X-ray absorptiometry (DXA). The authors stated that the individuals with CdLS had normal adiposity and low levels of lean mass measured with DXA [25]. These data are not consistent with ours, where the most common deviation was excessive FM in the whole study group. However, our results are not directly comparable with this study as BIA and DXA measurements differ. DXA delivers results in a three-compartment model and estimates fat mass, lean body mass and bone mineral content. The method utilizes the fact that the energy of an X-ray or photon beam is altered by the thickness, density, and chemical composition of an object in its path [9]. One must note that LBM (lean body mass) derived from DXA should not be described as FFM or compared to FFM derived from bioimpedance (BIA) measurements. They are not interchangeable as LBM consists of FFM plus the essential fat, which may consist of between 2 and 10% FFM. Moreover, lean mass measured by DXA may be compared to FFM minus bone minerals [26]. DXA is considered to be the reference standard for body composition measurements [27]. Nevertheless, BIA estimates body composition well when compared to standard methods, which was indicated in the Guidelines for the Evaluation and Treatment of Gastrointestinal and Nutritional Complications in Children With Neurological Impairment provided by the European Society for Paediatric Gastroenterology, Hepatology and Nutrition [28].

The InBody S10 analyzer used in our project is advanced and, according to the manufacturer’s instructions, it does not use empirical equations or a hydration factor. Conventional analyzer producers introduce various algorithms for calculating the hydration factor by age or gender to improve the measurements’ accuracy. In this way, they want to prevent false results associated with the assumption of BIA that the water content of FFM is constant but varies in reality [8]. Unlike conventional analyzers using low-frequency current, the InBody S10 uses multi-frequency current. Therefore, it measures both intracellular water (using a high-frequency current above 200 kHz) and extracellular water (using a low-frequency current below 50 kHz), which allows you to notice even discrete changes in hydration. Conventional analyzers predict the intracellular water based on extracellular water measurement and calculations.

We found the occurrence of dysphagia in 28.57% of our patients, with dysphagia defined as a PEDI-EAT-10 score equal to or greater than 3. This cut-off point resulted from the normative data suggesting that an EAT-10 score of 3 or higher is abnormal [29]. Some authors use a score ≥ 5 as the threshold to define dysphagia [4]. Practically, in our results, there were no patients who scored 3 or 4 points in PEDI-EAT-10, so our data confirm the assumptions of the publications cited above. The prevalence of dysphagia in our study reflects the present status of our patients, which may result from medical interventions in the past. Therefore, the frequency we observed seems to be much lower than even in the general population, in which the combined prevalence estimate of dysphagia is 60% [30].

Our work was based on data collected using a questionnaire. The patients did not come from a single medical center, but they were dispersed throughout the country, and they had received gastroenterological care in different centers by different doctors in the past. The data on past diagnoses were based on the declarations of the caregivers, in most cases without the possibility to consult the records. For this reason, we were not able to fully differentiate the causes of dysphagia in our study. We do not know the prevalence of eosinophilic esophagitis, food allergies or *Helicobacter pylori* infection in our study group. Some patients gave the impression that they were underdiagnosed, and we recommended they see their physicians in the place where they lived.

Our observations raise the topic of other causes of dysphagia in CdLS, which are different depending on age. In the general population, dysphagia in children might be caused by premature birth, low birth weight, developmental delay, neuromuscular disorders, anatomic abnormalities and cardiopulmonary disease [31]. In the middle age, dysphagia may be associated with gastroesophageal and immunologic causes. In older people, dysphagia may be a result of neoplastic and neurological diseases [32]. Indeed, the list of causes includes many that feature in CdLS, as the patients present neurodevelopmental delays or microcephaly. Additionally, a preterm birth and a history of tube feeding, common in the early life of CdLS patients, may play a role. In our study group, two patients from the dysphagic group had been tube fed in their first months of life, the patient coded as D4 up to six months old and the patient coded as D5 for 2 years due to cleft palate. There was another patient in the initial CdLS group who had been PEG tube fed until she was 4 years old. Her PEDI-EAT-10 score was 19, so she would have been included in the dysphagic group but she was excluded due to her anthropometric parameters being outside the range of the BIA analyzer.

GERD complications, such as erosive esophagitis or esophageal stricture, may manifest as dysphagia. Since PPIs were introduced in the 1990s, the incidence of esophageal stricture has been low and decreasing [33]. However, it may occur in CdLS patients even if they receive PPI therapy or after surgical treatment. Our initial group included a 27-year-old woman, who was excluded from the study because of a history of fundoplication at 6 months old. Her PEDI-EAT-10 score was 20; she was partially fed orally but mostly through a PEG tube. She suffered from severe, ongoing dysphagia symptoms probably due to esophageal stricture. On the other hand, surprisingly, according to a metanalysis conducted on a different group, patients with esophageal atresia after surgery showed no relationship between esophageal strictures and dysphagia [34]. These authors concluded that since many other factors contribute to dysphagia, comprehensive information such as detailed standardized registry systems for rare diseases for pooling analysis is needed to investigate other potential factors including surgical complications.

It should be noted that one patient from the dysphagia group (D5) had a history of aspiration pneumonia that required hospitalization. There were also three others—excluded from the study due to low height and thus not being suitable for BIA analysis, as well as a mistake with the height measurement—with PEDI-EAT-10 scores of 16, 19 and 19, and these patients had also had aspiration pneumonia. Moreover, all four had been diagnosed with GERD. In the non-dysphagic group, there was only one patient (ND7) who had been hospitalized due to pneumonia; however, it was a non-aspiration disease. These data are consistent with the available literature. Firstly, in children with neurological impairment, a linear correlation was found between PEDI-EAT-10 and PAS (The Penetration and Aspiration Scale) scores, which assesses the severity of penetration and aspiration [35]. Secondly, it has been reported that adult patients with an EAT-10 score > 15 are 2.2 times more likely to aspirate and that there is a linear correlation between the EAT-10 score and aspiration events and aspiration risk [36].

In addition to the data presented above, we calculated FMI for the patients, with the assumption that it could better describe their status. These data are presented in Table 2 and Table 3; however, we did not compare these results to norms or match them to centile grids. Comparing our data to norms for the healthy population appears unwarranted, as index calculations depend on height, which is genetically lower in CdLS. While the CdLS population has its own body height charts [23], neither BMI nor FMI norms have been established so far.

The literature contains studies regarding body composition and dysphagia in other conditions. An association between tongue pressure and factors related to sarcopenia such as aging, activities of daily living, nutritional state and dysphagia have been evaluated in the elderly, with the results suggesting that the clinical condition of sarcopenic dysphagia may be partially interpreted as the presence of sarcopenia and causal factors for sarcopenia [37]. In our group, five patients were deficient in SMM (skeletal muscle mass), including two from the dysphagia group and two from the non-dysphagic group. In a cross-sectional study such as this, it is not possible to determine what is an effect and what is the cause, as the results present associations only.

Cheney et al. [38] evaluated the relationship between body composition and dysphagia in patients with Amyotrophic Lateral Sclerosis (ALS). Positive associations were found between body compartments and swallowing analysis. A significant correlation between tongue pressure and body weight, FFM, SMM and trunk FFM was found. The phase angle showed a strong correlation with the Functional Oral Intake Score (r = 0.74, *p* < 0.01), Swallowing Rating Scale of the American Speech-Language-Hearing Association score (r = 0.77, *p* < 0.01) and tongue pressure. Another study involving institutionalized older adults showed that low PhA (<3.5°) was independently associated with the presence of oropharyngeal dysphagia, evaluated with a volumeviscosity swallow test [39]. In our original group, only one patient had a PhA below 3.5°, an 18-month-old girl with a PEDI-EAT-10 score of 16, and had to be excluded due to the working range of the analyzer and a possible error. Other authors stated that the optimal phase angle cutoff value to detect sarcopenia in older adults was ≤4.55° [16] or, in a different study, 4.05° for men and 3.55° for women [40]. A study conducted among 18-year-old students reported the value of PhA for sarcopenia in young male participants at 5.95 and in young female participants at 5.02° [18]. In our study group, all dysphagic patients and six of the non-dysphagic patients met these criteria. A high phase angle has also been reported as being related to increased BCM values and better overall health conditions [20]. Similar observations were made in our study, where children without dysphagia had normal or above-normal BCM and patients with dysphagia had normal or below-normal BCM. Additionally, the non-dysphagic group had higher PhA values than the dysphagic patients.

The small sample size is a limitation of our study. CdLS is a rare disease, and it is difficult to collect a large amount of data at once. However, treating our results as preliminary might motivate planning of further research in this area to collect data from a larger group that could be statistically analyzed. Moreover, during the BIA analysis, keeping some of the children still was difficult due to behavioral disorders. We excluded from the analysis the results obtained from children who moved vigorously. Repeating the measurement was not feasible due to difficulties with the children’s cooperation. In addition, some patients had anatomical defects in their fingers, hands, or whole limbs. No BIA analyzer is perfectly adapted to such a group, but it seemed reasonable to simply use adhesive electrodes for the limb that was present or apply them to finger stumps.

## 5. Conclusions

Dysphagia and abnormalities in body composition parameters are common among CdLS patients and this group requires nutritional and gastroenterological evaluation. The most prevalent are abnormalities in FM, both excess and deficit. PhA deviations observed in the bioimpedance study deepen with the severity of dysphagia. These preliminary data were collected in a small sample of patients with rare disease, and the findings require further investigation on a larger cohort of patients.

This study was approved by the relevant research ethics committee and informed consent was obtained from the research subjects.

## Figures and Tables

**Table 1:** Sex, age, genetic mutation, height and weight with their centile channels specific for CdLS according to Kline [23]. ND—non-dysphagic; D—dysphagic. M—male; F—female.

Patients Code	Gender	Age	Mutation	Body Height	Centile Channel for Height	Body Weight	Centile Channel for Weight
ND1	M	13	NIPBL	145	95	37	50–95
ND2	F	9.5	-	94	5–50	12.5	5–50
ND3	F	30	NIPBL	153	-	102.5	-
ND4	F	8.5	SMC3	112	50–95	19	50–95
ND5	M	12	NIPBL	130	50–95	24	50
ND6	F	6	NIPBL	107	50–95	22	>95
ND7	M	21	NIPBL	152	-	48.5	-
ND8	F	12	DDX23	146	>95	58	>95
ND9	F	42	NIPBL	145	-	53.5	-
ND10	F	42	NIPBL	145	-	72	-
D1	F	5	SMC1A	115	>95	21.7	>95
D3	M	6	NIPBL	105	50–95	15	50–95
D4	F	26	NIPBL	126.5	-	35	-
D5	M	14	NIPBL	108	<5	14.5	<5

**Table 2:** Characteristics of the dysphagia group. All norms refer to InBody analyzer data for each individual. N: within norm, ↓: below the normal range, ↑: above the normal range. GERD: patient has been diagnosed in the past with the disease. IPP: patient treated at the time of the study or treated when symptoms exacerbate. FFM: fat free mass. PhA: phase angle. BCM: Body Cell Mass. SMM: skeletal muscle mass. FM: fat mass. FMI: Fat mass index. D: dysphagic. M: male. F: female.

Patients Code	PEDI-EAT-10	GERD	IPP	FFM	PhA	BCM	SMM	FM	FMI	Age	Gender
D1	20	-	-	N	3.8	N	N	N	3.79	5	F
D3	5	-	-	N	4.3	N	N	↓	0.45	6	M
D4	10	-	-	↓	4.7	↓	↓	↑	9.55	26	F
D5	8	Yes	Yes	↓	4.2	↓	↓	↓	1.85	13.5	M

**Table 3:** Characteristics of the patients with PEDI-EAT-10 questionnaire score < 3. All norms refer to InBody analyzer data for each individual. N: within norm, ↓: below the normal range, ↑: above the normal range. GERD: patient has been diagnosed in the past with the disease. IPP: patient treated at the time of the study or treated when symptoms exacerbate. FFM: fat free mass. PhA: phase angle. BCM: Body Cell Mass. SMM: skeletal muscle mass. FM: fat mass. FMI: fat mass index. ND: non-dysphagic. M: male. F: female.

Patients Code	PEDI-EAT-10	GERD	IPP	FFM	PhA	BCM	SMM	FM	FMI	Age	Gender
ND 1	0	-	-	↑	4.9	N	N	↓	1.52	12.5	M
ND 2	1	Yes	-	N	4.1	N	↓	N	2.38	9.5	F
ND 3	1	Yes	Yes	↑	6.3	↑	↑	↑	22	30	F
ND 4	1	Yes	Yes	N	4.5	N	N	N	2.47	8.5	F
ND 5	0	Yes	Yes	N	5.5	N	N	↓	0.41	11.5	M
ND 6	2	Yes	-	N	4.5	N	↓	↑	7.34	6	F
ND 7	0	Yes	-	N	5.6	N	N	N	3.94	21	M
ND 8	0	-	-	N	5.3	N	N	↑	12.4	12	F
ND 9	0	Yes	Yes	N	6.3	N	N	↑	9.13	42	F
ND 10	0	Yes	-	N	5.9	N	N	↑	14.8	42	M

## Data Availability

The data presented in this study are available on request from the corresponding author.
